# Clinical outcomes of prophylactic compression sutures for treatment of uterine atony during the cesarean delivery of twins

**DOI:** 10.1186/s12884-019-2716-6

**Published:** 2020-01-16

**Authors:** Mi-La Kim, Yoon-Mi Hur, Hyejin Ryu, Min Jin Lee, Seok Ju Seong, Joong Sik Shin

**Affiliations:** 1 Department of Obstetrics and Gynecology, CHA Gangnam Medical Center, CHA University, 566, Nonhyeon-ro, Gangnam-gu, Seoul, 06135 Republic of Korea; 2 Department of Education, Institute for Education Research, Mokpo National University, Jeonnam, Republic of Korea

**Keywords:** Twin pregnancy, Uterine atony, Compression suture, Postpartum hemorrhage

## Abstract

**Background:**

Twin pregnancy has a high risk for developing uterine atony (UA). This study aimed to evaluate efficacy and clinical outcomes of prophylactic compression sutures to treat UA during twin cesarean section (CS).

**Methods:**

All patient records of twin deliveries by CS after gestational age of 24 weeks in a large maternity hospital in South Korea between January 2013 and June 2018 were reviewed. Patients with monochorionic monoamniotic twins were excluded from data analysis. In total, 953 women were eligible for data analysis.

**Results:**

Of the 953 patients, compression sutures were applied to 147 cases with postpartum bleeding that were refractory to uterine massage and uterotonics. Out of the 147, two patients (1.4%) proceeded to additional uterine artery ligation to achieve hemostasis, yielding a success rate of 98.6%. The rate of transfusion after the first 24 h of delivery in the suture group was not significantly different from that in the non-suture group, suggesting that both groups achieved hemostasis at an equal rate after the first 24 h of delivery. The difference in the operation time between the two groups was only 8.5 min. The rate of subsequent pregnancy among the patients who received compression sutures was 44.4%.

**Conclusions:**

Overall, our findings suggest that with early and fast implementation of compression sutures, UA can be treated in the setting of twin cesarean delivery without significantly increasing maternal morbidity.

## Background

Uterine atony (UA) is defined as failure of the uterus to contract adequately during and after the third stage of labor [1]. Normally, uterine muscles contract during labor and immediately after the placenta is delivered to compress the blood vessels. The compression of the blood vessels reduces the blood flow, and thereby increases the likelihood of coagulation and prevents postpartum hemorrhage, which is one of the leading causes of maternal morbidity and mortality worldwide [2, 3]. UA is the most common cause of postpartum hemorrhage: up to 80% of postpartum hemorrhage cases have been reported to be related to UA [4]. Twin pregnancy is known to be a risk factor for developing UA, although other factors such as overdistension of the uterus, labor abnormalities, obesity, and previous history of postpartum hemorrhage are also known to contribute to UA [5, 6].

The first step in managing UA is application of nonsurgical therapies including uterine massage, uterotonics, and manual compression. If these nonsurgical methods do not achieve hemostasis, surgical approaches must be considered as a next step. Surgical options include uterine compression sutures, balloon tamponade, uterine artery or internal iliac artery ligation, uterine artery embolization, or hysterectomy. Since the publication of the B-Lynch compression suture in 1997 [7], various techniques of uterine compression sutures have been introduced and used worldwide as safe alternatives to other surgical methods [8–12]. Uterine compression suture techniques are simple and effective to treat UA, while preserving the anatomical integrity of the uterus and thus future fertility in many women. A review article reported that the rates of subsequent pregnancy among patients who had uterine compression sutures were 11–75%, with an average of 32% [13].

In the past two decades a number of studies demonstrated merits and the efficacy of uterine compression sutures to treat UA [4, 9]. However, most of these studies were on the basis of a sample of women with singleton deliveries [14] or a mixed sample of women with singleton and twin deliveries [9, 11]. In spite of the fact that twin pregnancy has a high risk for developing UA, to our knowledge, none of the studies to date examined the efficacy of UA in twin deliveries alone. The main goal of this study was therefore to evaluate the efficacy and clinical outcomes of the prophylactic compression sutures in the patients of UA who delivered twins by cesarean section (CS). To this end, we reviewed patient records in a large maternity hospital in Seoul, South Korea, where a B-Lynch suture modified by the last author of this report (JSS) and a multiple square suture by Cho (hereafter, Cho’s suture) [8] were routinely used to treat UA during CS.

## Methods

### Patient characteristics and clinical definitions

Patient records included data of the patient’s age at delivery, parity, mode of conception, gestational age at delivery, a sum of birthweight of the two members of a twin pair (sum of birthweight), history of previous abdomino-pelvic surgery, operation time (from skin incision to skin closure), estimated intraoperative blood loss, pre- and postoperative hemoglobin change, postpartum hemorrhage (over 1000 ml of blood loss with signs of hypovolemia) [15], whether or not transfusion was given, the timing of transfusion, and whether and what additional procedures were used to control the bleeding. Subsequent pregnancy outcomes were available for a subsample of patients.

We analyzed data for twin deliveries by CS after gestational age of 24 weeks that occurred in our institution from January 2013 to June 2018. Patients with monochorionic monoamniotic twins were excluded from data analysis. Cases with vaginal deliveries of twins were eliminated because usually, intrauterine balloon tamponade or uterine artery embolization were considered for treatment of UA in these patients in our institution. In our study, failure of management was defined as the need to proceed to other surgical methods such as uterine artery ligation, embolization, or hysterectomy after application of the compression suture. This study was approved by the institutional review boards of the CHA Gangnam Medical Center, Seoul, Republic of Korea. Data were anonymized and de-identified before analysis, and therefore, informed consent was not required.

Immediately after the delivery of twins, 20 units of prophylactic oxytocin mixed with main fluid were infused and 100mcg of carbetocin was injected intravenously as routine procedures. The bimanual uterine compression and massage were continuously given to the patients by surgeon or assistant. After 2 min (considering the onset of action of carbetocin) 1 mg of sulprostone mixed with normal saline (500 ml) was additionally administered in case of poor uterine contraction. The decision of whether to perform prophylactic compression suture or not was followed on the basis of both the degree of uterine contractility and the amount of bleeding within 4 to 5 min, which is the time required for the onset of action of sulprostone. Thus, it usually took 5–10 min for surgeons to make a diagnosis before they place compression sutures to patients.

### Methods of modified B-Lynch sutures in our institution

**Fig. 1:**
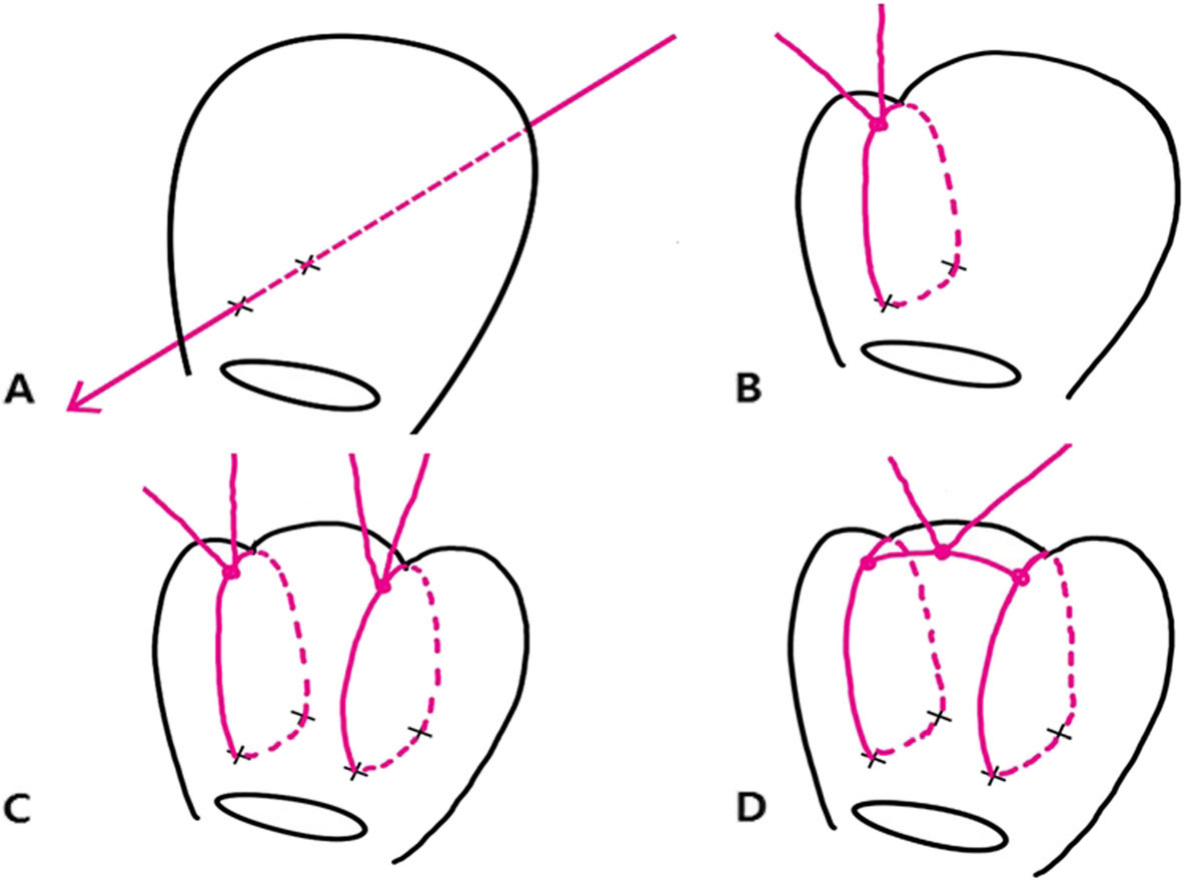
The modified B-Lynch uterine compression suture. **a** The straight needle was passed through the lateral side of the uterus from the posterior to anterior walls, 2~3 cm superior from the upper edge of the low flap transverse incision. **b** The vertical brace suture was tied at the fundus with compression tightly. The end edge of the suture tie was tagged with Kelly clamps. **c** The same procedure was repeated on the contralateral side. **d** At the fundus of the uterus, both tagged sutures were tied together at the midline to prevent slippage of the sutures

**Fig. 2:**
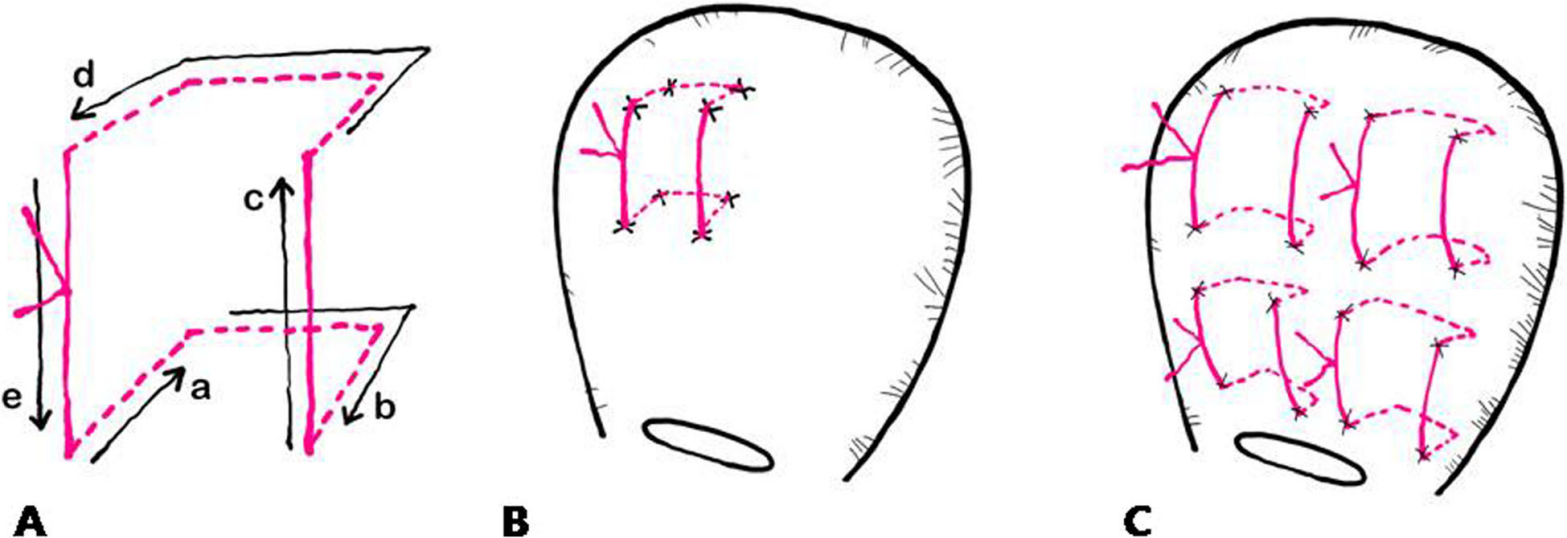
The Cho’s square suture technique. **A**. The straight needle was passed through bleeding sites to compress the endometrial cavity; firstly it was passed from the anterior to posterior walls (a), and 2~3 cm laterally, the needle was passed back through posterior to anterior walls (b), then, the needle was moved 2~3 cm upwards (c), and the same procedure was repeated in the opposite direction (d). The knot was tied as tightly as possible (e). **B**. Squared suture was completed. **C**. Multiple squared sutures by Cho’s suture can be used for uterine atony after twin cesarean delivery

Vicryl No. #1 sutures and straight needle No. 7 are used in the modified B-Lynch suture technique in our institution. The procedures are shown in Fig. 1. The incision site is closed when the uterine contraction is recovered and the amount of bleeding decreases. While the lead surgeon is performing the suture, the assisting surgeon performs bi-manual uterine compression. A video of the procedures is attached as an Additional file 1: Video S1.The Cho’s suture technique also uses Vicryl No. #1 sutures and straight needle No. 7. The details of the Cho’s suture method describe in Fig. 2.

### Statistical analysis

Statistical analysis was conducted using Statistical Package for Social Sciences (SPSS) version 22. The Chi-square and Fisher’s exact tests were used for analysis of categorical variables, and the Student’s t-test and Mann-Whitney U test were used for quantitative variables. A *p*-value of < 0.05 was considered statistically significant.

## Results

### Baseline characteristics of the patients and perioperative findings

During the 5 years of the study period, a total of 953 cases of twin cesarean deliveries were performed. Of these, either modified B-Lynch or Cho’s suture technique was applied to 147 patients because their uteri did not respond to massage and uterotonics. Modified B-Lynch suture was usually performed in case of poor uterine contraction, and Cho’s suture technique was applied to uterine atony along with placenta previa or accreta, and in some cases, in addition to modified B-Lynch suture. Table 1 shows the baseline characteristics of the patients by application status of the compression suture (patients without compression sutures versus patients with compression sutures). No statistically significant difference was found between the two groups in terms of patients’ age at delivery, parity, mode of conception, previous history of abdomino-pelvic surgery, or chorionicity. However, maternal body mass index (BMI) at delivery was significantly higher in patients with compression sutures as compared to those without compression sutures (27.5 vs. 26.9; *p* = 0.039), which was consistent with prior studies showing that obesity is a risk factor for developing UA [1, 16].

**Table 1:** Baseline characteristics of patients

Characteristics	Patients without compression sutures (*n* = 806)	Patients with compression sutures (*n* = 147)	*p*-value
Age at delivery (year)^a^	34.3 ± 3.1	34.0 ± 3.1	0.349
BMI at delivery (kg/m^2^)^a^	26.9 ± 3.2	27.5 ± 3.3	0.039
Previous abdomino-pelvic surgery^b^	265 (32.9%)	50 (34.0%)	0.776
Mode of conception^b^			0.150
Natural	77 (9.5%)	11 (7.5%)	
COH + TI/IUI	99 (12.3%)	11 (7.5%)	
IVF/T-ET	630 (78.2%)	125 (85.0%)	
Parity^c^			0.175
0	699 (86.7%)	134 (91.2%)	
≥ 1	107 (13.3%)	13 (8.8%)	
Chorionicity^c^			0.25
MCDA	69 (8.6%)	8 (5.4%)	
DCDA	737 (91.4%)	139 (94.6%)	

*BMI* Body mass index, *COH* Controlled ovarian hyperstimulation, *TI* Timed intercourse, *IUI* Intrauterine insemination, *IVF* In vitro fertilization, *T-ET* Thawing-embryo transfer, *MCDA* Monochorionic diamniotic, *DCDA* Dichorionic diamniotic. N (%) or mean ± standard deviation

^a^Student’s t-test, ^b^Chi-squared test, ^c^Fisher’s exact test

Table 2 shows the perioperative findings during CS of twins. Sum of birth weight was significantly larger in patients with compression sutures as compared to those without compression sutures (*p* = 0.018). Not unexpectedly, as a result of poor uterine contraction, the group with sutures was significantly higher than the group without sutures in estimated blood loss, hemoglobin change, and the rates of postpartum hemorrhage and transfusion within the first 24 h of delivery. However, the rate of transfusion after the first 24 h of delivery in the suture group was not significantly different from that in the non-suture group; both groups achieved hemostasis at an equal rate after the first 24 h of delivery. Of 147 who were treated with compression sutures, only two patients (1.4%) proceeded to the uterine artery ligation procedure additionally to achieve hemostasis, which resulted in a success rate of 98.6% of the uterine compression suture. As expected, operation time was significantly longer in the suture than in the non-suture group due to the application of sutures; however, the mean time difference was only 8.5 min.

**Table 2:** Perioperative findings

Characteristics	Patients without compression sutures (*n* = 806)	Patients with compression sutures (*n* = 147)	*p*-value
Gestational age at delivery (weeks)^a^	36.0 ± 2.1	36.3 ± 1.6	0.874
Birth weight (gm)^a^	4783.1 ± 832.8	4958.0 ± 768.4	0.018
Emergency delivery^b^	348 (43.2%)	68 (46.3%)	0.527
Operation time (min)^a^	51.3 ± 13.2	59.8 ± 17.7	< 0.001
Estimated blood loss (mL)^a^	611.9 ± 166.4	725.2 ± 256.1	< 0.001
Hemoglobin change^a^	2.0 ± 1.2 (*n* = 803)	2.4 ± 1.4 (*n* = 142)	0.001
Postpartum hemorrhage^b^	41 (5.1%)	28 (19.0%)	< 0.001
Transfusion during admission^b^	60 (7.4%)	27 (18.4%)	< 0.001
Within 24 h transfusion^b^	40 (5.0%)	22 (15.0%)	< 0.001
> 24 h transfusion^b^	24 (3.0%)	5 (3.4%)	0.793
Additional procedures needed to control the bleeding^b^	5 (0.6%)	2 (1.4%)	0.295

^a^Student’s t-test, ^b^Fisher’s exact test; N (%) or mean ± standard deviation

In the non-suture group, five patients (0.6%) were treated with other procedures. One patient was treated by uterine artery ligations during CS because the uterine incision was extended to the left uterine artery. To control the bleeding, multiple ligations of left uterine artery were performed. The remaining four patients had late-onset UA, and therefore, were transferred for uterine artery embolization after 24 h of delivery.

### Pregnancy outcomes following sutures

None of the patients with the compression suture developed complications related to the procedure during the first 2 months postpartum period. Among 953 patients, 371 (39%; *n* = 292 for the patients without sutures; *n* = 79 for the patients with sutures) were available for the follow-up investigation after the first 2 months postpartum period. Of these, 308 (*n* = 238 for the patients without sutures; *n* = 70 for the patients with sutures) expressed no desire to become pregnant, 22 (*n* = 17 for the patients without sutures; *n* = 5 for the patients with sutures) have tried to conceive, and 41 (*n* = 37 for the patients without sutures; *n* = 4 for the patients with sutures) became pregnant. The high rate (83% = 308/371) of giving up future pregnancies in our follow-up sample is probably a reflection of the very low birth rate in current Korean society [17]. In addition, since our patients had twins, and most of our patients were somewhat old aged for another childbirth (see Table 1), these factors possibly contributed to low interests in subsequent pregnancies. Excluding patients who did not desire future pregnancies, the rate of subsequent pregnancy among those with sutures in our follow-up sample was 44.4% (= 4/9), which was within the range reported in the literature [13]. However, given that our follow-up sample only included 39% of the original sample, as well as the fact that the vast majority of the follow-up sample had no interests in future pregnancy, and that the patients in our study were on the basis of the most recent 5 years’ records in our institution, our rate of future fertility should be considered only as an approximate estimate.

Table 3 shows a summary of pregnancy outcome data for the 41 patients sub-grouped according to the application status of the compression suture. As only four of the 41 patients received compression sutures, we did not carry out statistical analysis to compare patients with and without compression sutures in pregnancy outcomes. However, the mean intervals between CS and subsequent pregnancy were very similar in between the two groups (21.2 months for the patients without sutures and 22.5 months for the patients with sutures), suggesting that the adverse effect of sutures on future pregnancy may be minimal. Among the four patients with sutures, one received assisted reproductive technology (ART) and had ongoing pregnancy, and the remaining three conceived spontaneously, of which one had a full-term delivery, and two had been in ongoing pregnancy.

**Table 3:** A summary of pregnancy outcomes of the patients with and without compression sutures

Characteristics	Patients without compression sutures (n = 37)	Patients with compression sutures (n = 4)
Interval to subsequent pregnancy (months)	21.2 ± 13.2	22.5 ± 6.4
Mode of conception		
Natural	33(89.2%)	3 (75%)
ART	4 (10.8%)	1 (25%)
Subsequent delivery outcomes		
Full-term delivery	14 (37.8%)	1 (25%)
Preterm delivery	0 (0%)	0 (0%)
Abortion	7 (18.9%)	0 (0%)
Ectopic pregnancy	3 (8.1%)	0 (0%)
Ongoing pregnancy	13 (35.2%)	3 (75%)

*ART* Assisted reproductive technology, *C/S* Cesarean section; n(%) or mean ± standard deviation

## Discussion

To our knowledge, this is a large cohort study on clinical outcomes of uterine compression suture performed under the institutional guideline in cesarean delivery of twins. Our results demonstrated that compression sutures for UA in CS of twins were effective (98.6% success rate) and they are easily implementable techniques that required only 8.5 min of additional operation time on average. A recent review of studies of compression sutures for UA yielded an average success rate of 97% (range: 76 to 100%) [13], while an earlier review showed 91.7% [18]. Despite that our sample had generally higher obstetric risks associated with twin deliveries [19], our success rate was higher than the average success rates found in cesarean deliveries of singletons. Furthermore, although cesarean delivery is a high-risk factor for hysterectomy [20], none of our study sample underwent hysterectomy.

Although gestational age at delivery was not significantly different between the two groups, sum of birthweight was significantly higher in patients with sutures than those without sutures (4958.0 versus. 4783.1 g, *p* = 0.018), which appeared to be due to larger BMI at delivery in patients with sutures than those without sutures because BMI is a highly heritable trait [21, 22].

In Kaya et al.’s study, Bakri balloon tamponade, as a less invasive uterine preserving method, was compared with B-Lynch compression suture [23]. In their prospective study, the success rates, mean duration of time to bleeding control, estimated blood loss, numbers of transfused packed red blood cells were similar in both methods. However, the duration of operation was significantly longer in the Bakri balloon tamponade group [23]. Considering the aspects of application time, we proposed Shin’s modified B-Lynch suture as a primary uterus-sparing method in cases of uterine atony without response to medical treatment during CS. B-Lynch suture needs incision of the lower uterine segment (like a CS incision). In cases of suspected uterine atony after the uterine incision was closed already, the incision site must be opened before the application of B-Lynch suture. Additionally, B-Lynch suture cannot transfix the whole thickness of both uterine walls. However, modified B-Lynch suture in our institution is much faster and easier than B-Lynch suture and transfixes the uterine wall without slip down the brace. Cho’s suture technique, in addition, controlled focal bleeding with ease. We also agree that the Bakri balloon tamponade is the first line treatment in uterine atony during vaginal delivery. However, in this article, we analyzed in CS.

A major strength of our study is the inclusion of a large cohort of patients. However, this study has several limitations. The incidence of UA in our study sample (15.8% including the late onset cases) is higher than those found in other studies [11, 16]. This higher incidence may be due to the fact that our sample consisted of women with twin deliveries alone, while most of prior studies were based on women with singleton deliveries [9]. We also speculate that in order to prevent the development of UA, our surgeons made fast decisions and applied sutures to less severe cases of UA, which might have resulted in higher prevalence of UA in our sample. Additionally, generalization of our results may be limited because patients were from only a single hospital located in relatively high socioeconomic neighborhood in Seoul, Republic of Korea. Further large scaled studies are necessary to confirm the prevalence of UA in women with twin deliveries.

In a large British population-based sample, Kayem et al. [24] found that the success rate of compression sutures techniques depended on the time interval between delivery and uterine compression sutures; compression sutures performed within 1 h after delivery demonstrated a success rate of 84%, while a delay of between 2 and 6 h from delivery decreased the rate to be 58%. These results suggest that the high success rate in our study may be attributable to our surgeons’ fast diagnosis and immediate treatment. In our hospital surgeons typically spend additional 8.5 min for UA patients including diagnosis and applications of sutures. We noted from our experience that compression sutures should be applied before the occurrence of disseminated intravascular coagulation (DIC), that earlier implementation of sutures required fewer sutures, and that the possibility of tissue ischemia could be reduced with earlier implementation.

## Conclusion

In conclusion, our findings indicate that with early and fast implementation of compression sutures, UA can be treated in the setting of twin cesarean delivery without a significant increase maternal morbidity. Multicenter studies are needed to corroborate our findings.

## Supplementary information

**Additional file 1:** **Video S1.** Video that demonstrates the Shin’s modified B-Lynch sutures

## Data Availability

Data will be available upon reasonable request from the corresponding author. However, the data cannot be made public to maintain women’s privacy and legal reasons as it contains private health information along with identifiers.

## Abbreviations

ART: Assisted reproductive technology
BMI: Body mass index
CS: Cesarean section
UA: Uterine atony
